# Taichi on the brain: an activation likelihood estimated meta-analysis of functional neuroimaging data

**DOI:** 10.3389/fnhum.2024.1493677

**Published:** 2025-01-22

**Authors:** Shengxin Wang, Tianyu Liu, Jingtao Du, Jun Chen, Xiufen Luo, Yujie Meng, Chun Zeng, Xupeng Zhang, Binghua Shao

**Affiliations:** ^1^School of Physical Education, Chengdu Technological University, Chengdu, China; ^2^School of Physical Education and Health, Chengdu University of TCM, Chengdu, China; ^3^Department of Physical Education, Sichuan Vocational and Technical College of Communications, Chengdu, China

**Keywords:** Tai Chi Chuan, functional MRI, activation likelihood estimation, meta-analysis, neuroimaging

## Abstract

**Introduction:**

Tai Chi Chuan (TCC) is an exercise regimen renowned for its comprehensive benefits to both physical and mental health. The present research endeavor aims to elucidate the neurocognitive impacts of TCC compared to alternative exercise modalities or therapeutic interventions.

**Methods:**

A systematic meta-analysis was undertaken, encompassing a rigorous review of diverse datasets, wherein 422 scholarly articles were examined, with a subset of 18 articles meeting the stringent criteria for inclusion in the analytical framework.

**Results:**

The study cohort comprised 677 participants, characterized by a mean age of 56.52 ± 14.89 years and an average educational attainment of 11.06 ± 3.32 years. Noteworthy alterations in functional neural activity were identified within the superior frontal gyrus.

**Discussion:**

This comprehensive analysis provides significant insights into the enduring neural modifications and the distinctive contributions of TCC to cognitive health. Nevertheless, it is imperative to acknowledge the potential for bias in smaller functional magnetic resonance imaging studies owing to their inconclusive outcomes. This observation underscores the critical need for collaborative, multicenter research initiatives with expanded sample sizes to enhance the robustness and generalizability of future findings.

## Introduction

Tai Chi Chuan (TCC) is a holistic practice encompassing physical and mental dimensions, frequently utilized as a complementary and alternative therapeutic modality (Wayne et al., 2014). TCC comprises intricate movements integrating components such as squatting and deep breathing, purported to promote relaxation and alleviate pain (Kong et al., 2016). As of the conclusion of the year 2022, TCC has proliferated its reach to over 150 countries and regions, garnering a substantial global adherent base exceeding 400 million individuals (Yang et al., 2021). Numerous clinical trials and systematic reviews have been conducted to investigate the efficacy of TCC. The most recent Cochrane review on physical activity and chronic pain indicates that interventions such as TCC may effectively mitigate chronic pain and enhance overall quality of life (Geneen et al., 2017). Additionally, there has been an escalating emphasis on elucidating the central nervous system mechanisms underlying TCC to comprehensively understand its functional dynamics and efficacy from a neurological standpoint (Wang et al., 2022). However, these studies' quality, design, and outcomes have exhibited variability and need more robust and conclusive evidence.

The elucidation of CNS mechanisms constitutes a focal point of consensus and intensive inquiry within the burgeoning field of neuroscience research on TCC. Many researchers have investigated the mechanisms by which TCC enhances cognitive function, the long-term impacts of TCC exercise on the brain, and its effects on neurological disorders, examining these phenomena from structural and functional perspectives (Yu et al., 2018). TCC training has been demonstrated to effectively augment spontaneous functional neural activity and enhance participants' cognitive capacities, including memory, executive control, and emotional regulation. Functional magnetic resonance imaging (fMRI) serves as a pivotal tool, offering a visual and quantitative methodology for delineating the effects of TCC on the CNS. A rigorously designed fMRI study has the potential to yield robust neural activity features that can be instrumental in diagnostic, therapeutic, and machine-learning applications. Nevertheless, discrepancies in study designs, imaging acquisition protocols, preprocessing pipelines, and data analysis methodologies have precipitated heterogeneous findings, thereby complicating the synthesis of consistent conclusions.

To date, a comprehensive systematic evaluation of the impact of TCC on CNS remains lacking, and extant descriptive studies have not succeeded in accurately quantifying and summarizing the findings (Yu et al., 2018). An activation likelihood estimation (ALE) analysis represents the appropriate methodology for validating these effects as it facilitates the synthesis of all current studies and the derivation of consistent conclusions (Eickhoff et al., 2009). The ALE approach assesses the overlap of activation foci by modeling them as probability distributions centered on their respective coordinate locations (Turkeltaub et al., 2012). We posit that TCC exerts distinct neuromodulatory effects, manifesting variably among healthy individuals and those afflicted with specific conditions. These neuromodulatory influences are amenable to observation through diverse imaging methodologies. Moreover, different imaging modalities and analytical techniques can capture distinct facets of TCC's neuromodulatory impact. This hypothesis lends credence to the proposition that ALE constitutes the most appropriate investigative methodology. In contradistinction to prior MRI studies on TCC, the present investigation employs ALE analysis. ALE analysis is an imaging-based approach that diverges from conventional meta-analytic methods, which commonly integrate effect size metrics such as risk ratios. Instead, ALE analysis prioritizes the spatial coordinates of neuroimaging results, thereby more precisely evaluating the brain regions affected by TCC practice. This approach will delineate regions of interest pertinent to the neurological effects of TCC, thereby providing a reliable foundation for the design and analysis of future TCC research. Consequently, the primary objective of this study is to investigate the neural mechanisms underlying TCC and proffer recommendations for neuroimaging studies on TCC. This will be achieved through a systematic process encompassing standardized literature retrieval and screening, comprehensive literature evaluation, and application of ALE analysis.

## Methods

This report follows the Preferred Reporting Items of the Systematic Reviews and Meta-Analyses (Page et al., 2021) (PRISMA; Supplementary Table 1) and Neuroimaging Meta-Analyses Guidelines (Muller et al., 2018).

###  Literature search and study selection

**Figure 1:**
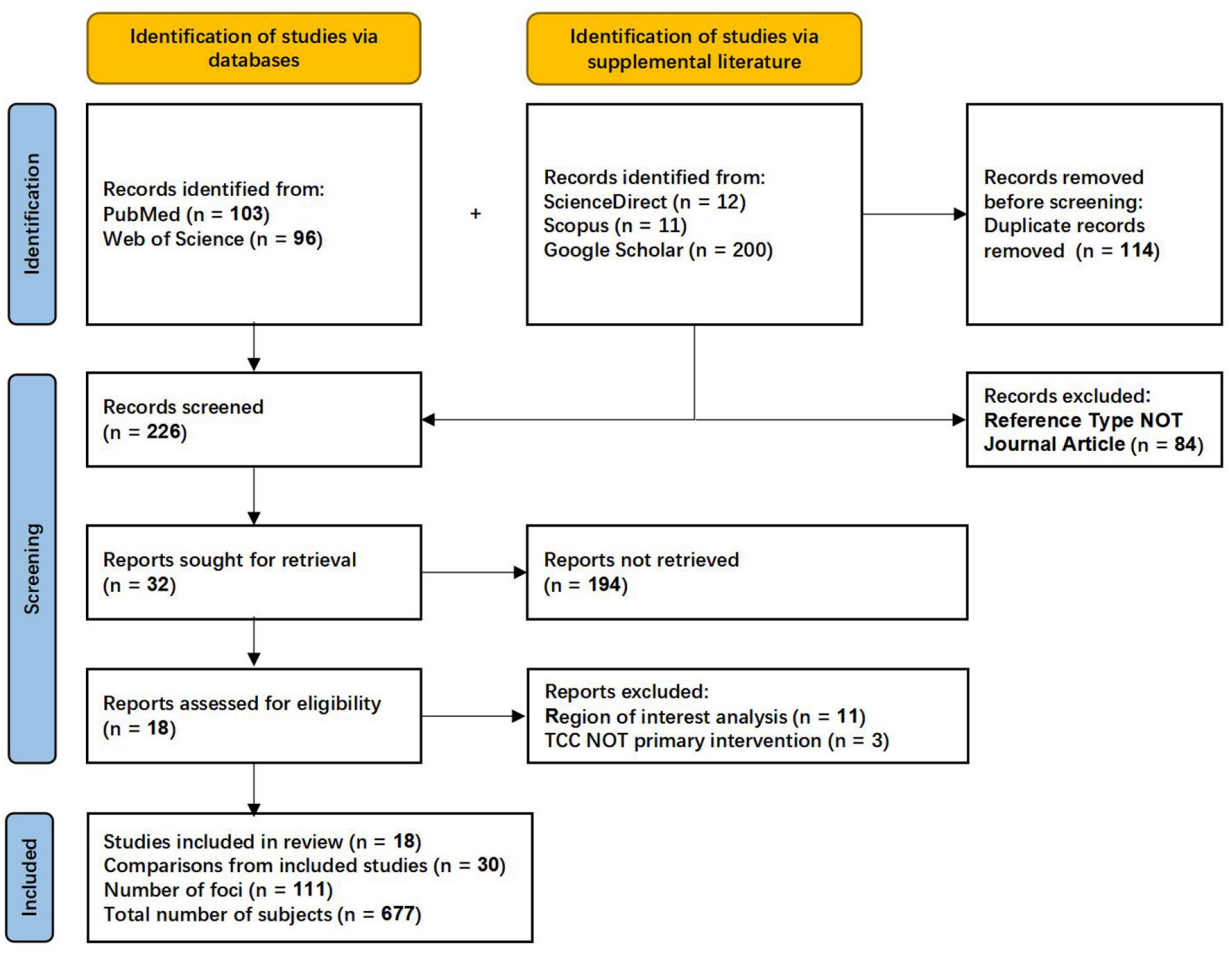
PRISMA flowchart of literature search and selection process.

To retrieve studies as comprehensively as possible, we used Medical Subject Headings (MeSH). Specifically: (1) the MeSH words “Tai Ji• and “Magnetic Resonance Imaging• were used for searching in PubMed; (2) all entry terms in the mesh words were used as topic terms for mesh-like searches in Web of Science; (3) the ScienceDirect, Scopus, and Google scholar as supplemental database, with the same search expression as Web of Science. The publication year was before 1 September 2024 (Figure 1). After removing duplicates, the preliminary studies library was filtered by title and abstract. We retained only studies meeting the following criteria: (1) research written in English; (2) original fMRI studies; (3) the study reports whole-brain activation coordinates, not region of interest (ROI) or small volume correction (SVC) results; (4) TCC as a primary intervention. Exclusion criteria: (1) studies that did not report certain required information (including number of subjects, age, coordinates); (2) no comparisons were reported for this study; (3) no significant results.

###  Data extraction

Starting with an initial screening of titles and abstracts, a comprehensive review of 32 potentially relevant papers was performed according to the above selection criteria (Figure 1). Extracted data were analyzed using the MNI coordinate system, and coordinate transformations were performed for studies using Talairach coordinates. Record retrieval, inclusion and exclusion, and coordinate extraction were independently completed by two researchers and then cross-validated. A third person was required to vote for records and data with objections.

###  Literature quality evaluation

Since fMRI research needs to focus more on image acquisition and processing, and there is no recognized quality evaluation method for fMRI research, existing tools such as Cochrane Risk of Bias tools (Minozzi et al., 2020) and GRADE (Viswanathan et al., 2018) cannot reasonably evaluate neuroimaging quality, so we use a more recognized evaluation method. A form is designed with ten questions on subject quality, research methods, image acquisition and analysis quality, results, and conclusion quality and uses 0, 0.5, and 1 for scoring. It can effectively quantitatively evaluate the research methods in fMRI research (Strakowski et al., 2000).

###  Publication bias

The prevalence of coordinate-based meta-analyses has been hampered by the exclusion of unpublished studies, often due to a lack of statistically significant findings (David et al., 2013). We used correlation analysis to ensure the robustness of ALE-analysis results to this publication bias by calculating the relationship between the number of participants and the number of reported significant findings (number of foci reported) (David et al., 2018). We consider the negative correlation between sample size and number of foci typical of analyses with publication bias, in which studies with small samples are published only when their results match the a priori hypothesis (Alegria et al., 2016). Based on the above assumptions, we will use Pearson's correlation analysis to verify the relationship between the sample size and the number of foci.

###  Activation likelihood estimation

We performed ALE analysis using GingerALE 3.0.2 software (Turkeltaub et al., 2012) to determine neurological effects of TCC. We found that multiple comparisons from the same set of subjects may create dependencies between experimental plots, thereby reducing the validity of the meta-analysis results. To prevent this problem, for each meta-analysis, we studied all coordinates of related contrasts, which were combined into one experiment to adjust for within-group effects. ALE scores were tested against ALE scores obtained under the null distribution with a threshold of *P* < 0.05, corrected for voxel-level family-wise error, and a minimal volume threshold set at volume >100 *mm*^3^. GingerALE automatically generated anatomical labels for all clusters. In addition, we also used the dpabi toolbox (Yan et al., 2016) for double-checking these localizations and the presentation of results.

## Results

###  General literature information

The search strategy resulted in 422 relevant articles, and 18 articles were included in this meta-analysis (Figure 1). These studies reported that resting state functional connectivity (rs-FC) changes the most. Six hundred and seventy-seven subjects were enrolled in the current review with 56.52 ± 14.89 years of age and 11.06 ± 3.32 years of education (Table 1; Supplementary Table 2).

**Table 1:** General literature information.

**Study ID**	**Sample size**	**Gender (male/female)**	**Age (years)**	**Education (years)**
Tao, J. 2016	21	8/13	62.38 ± 4.55	9.61 ± 3.02
Tao, J. 2017a	21	8/13	62.38 ± 4.55	9.61 ± 3.02
Tao, J. 2017b	21	8/13	62.38 ± 4.55	9.61 ± 3.02
Liu, Z. 2018	26	8/18	65.19 ± 2.30	10.46 ± 1.79
Wu, M. 2018	16	3/13	64.9 ± 2.8	13.8 ± 2.4
Cui, L. 2019	12	2/10	21.83 ± 2.48	16.33 ± 2.23
Kong, J. 2019	21	1/20	53.10 ± 11.58	N.A.
Liu, J. 2019a	28	6/22	40–70	N.A.
Liu, J. 2019b	28	6/22	40–70	N.A.
Liu, J. 2019c	21	8/13	62.38 ± 4.55	9.61 ± 3.02
Chen, L. 2020	22	7/15	52.36 ± 6.88	12.18 ± 3.03
Liu, Z. 2020	31	10/21	64.93 ± 2.37	10.52 ± 1.91
Xu, A. 2020	16	6/10	46.5 ± 18.5	N.A.
Yue, C. 2020a	20	0/20	62.90 ± 2.38	9.05 ± 1.80
Shen, Q. 2021	12	2/10	21.83 ± 2.48	16.33 ± 2.23
Shen, H. 2022	20	15/5	66.90 ± 7.17	N.A.
Zhang, J. 2023	9	2/7	24.20 ± 4.07	N.A.
Liu, J. 2024	28	6/22	40–70	N.A.

rs-FC, resting-state functional connectivity; ReHo, regional homogeneity; VMHC, voxel-mirrored homotopic connectivity; ALFF, amplitude of low-frequency fluctuation; N/A, not applicable.

###  Literature quality

**Figure 2:**
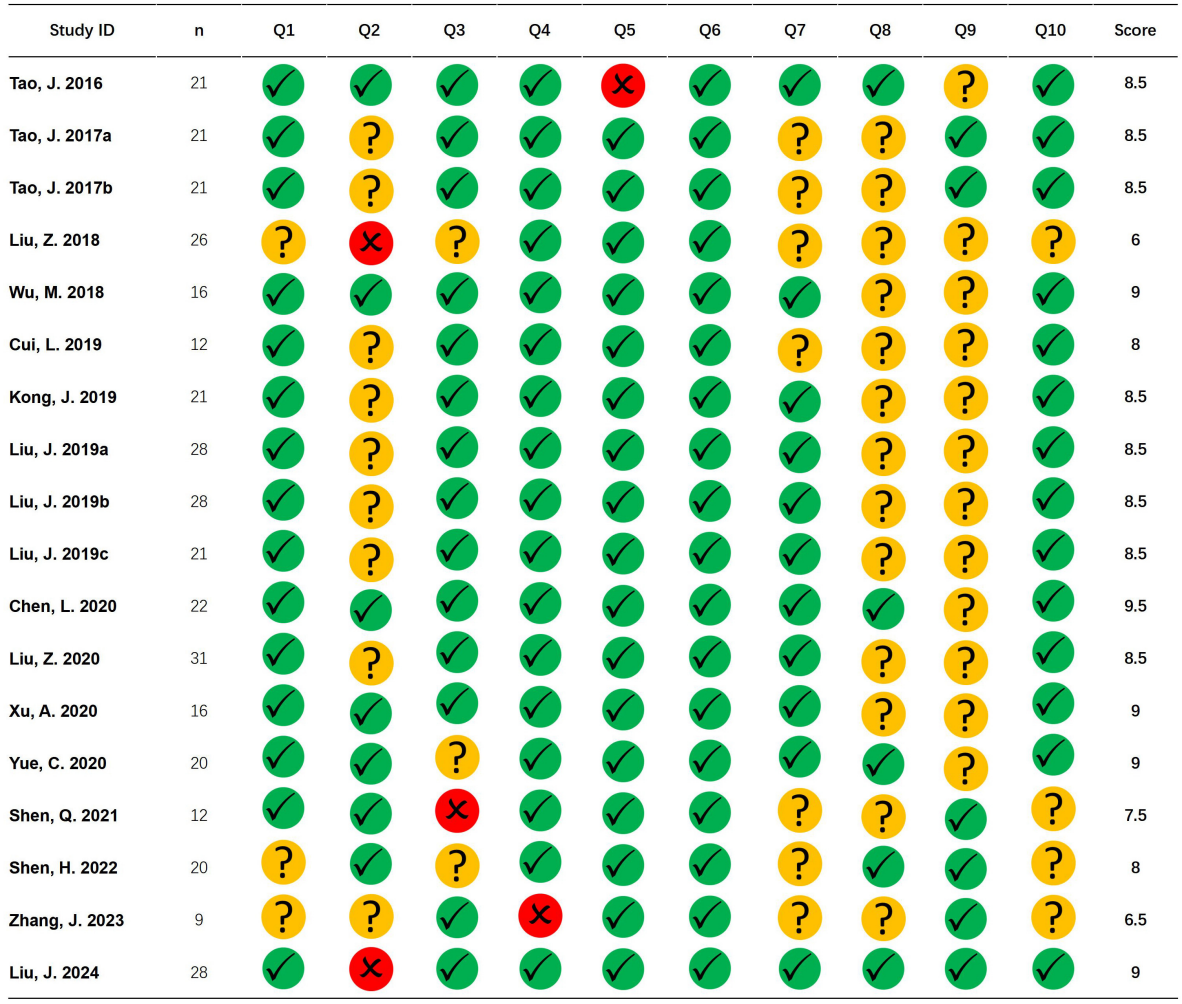
Literature quality. The figure shows the quality evaluation of the included studies. Green circle with checkmark represents the question with a score of 1, yellow circle with a question mark represents the question with a score of 0.5, and red circle with a cross represents the question with a score of 0.

The average total score of all 18 records was 8.306, with a low of 6 and a high of 9 for a single publication. Among all records, overall quality performed better on question 6 (standard space) and worse on question 2 (baseline information) and question 8 (multiple comparison correction; Figure 2).

###  Publication bias

**Figure 3:**
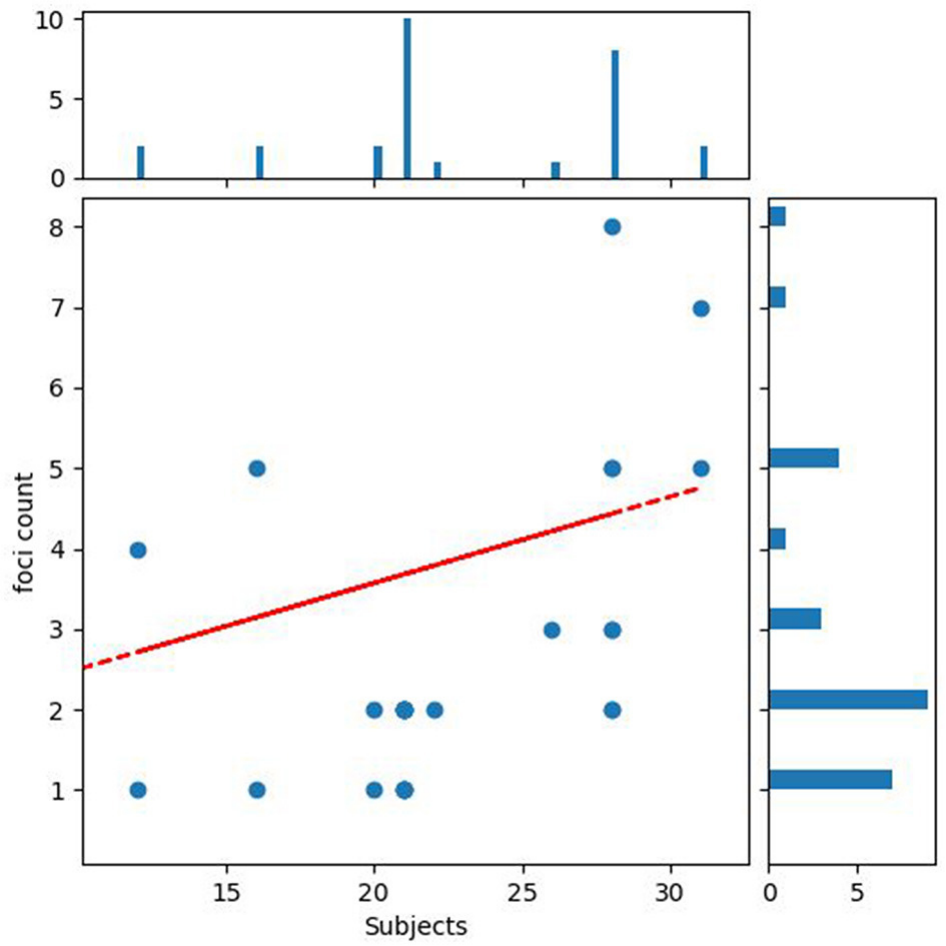
Publication bias. The figure shows the relationship between the number of participants and the number of reported significant findings.

Against this possible confound, for pooled ALE meta-analyses, we observed no significant negative correlation between sample size and number of foci action relational: *r* = 0.099, *P* = 0.609 (Figure 3).

###  Activation likelihood estimation

**Figure 4:**
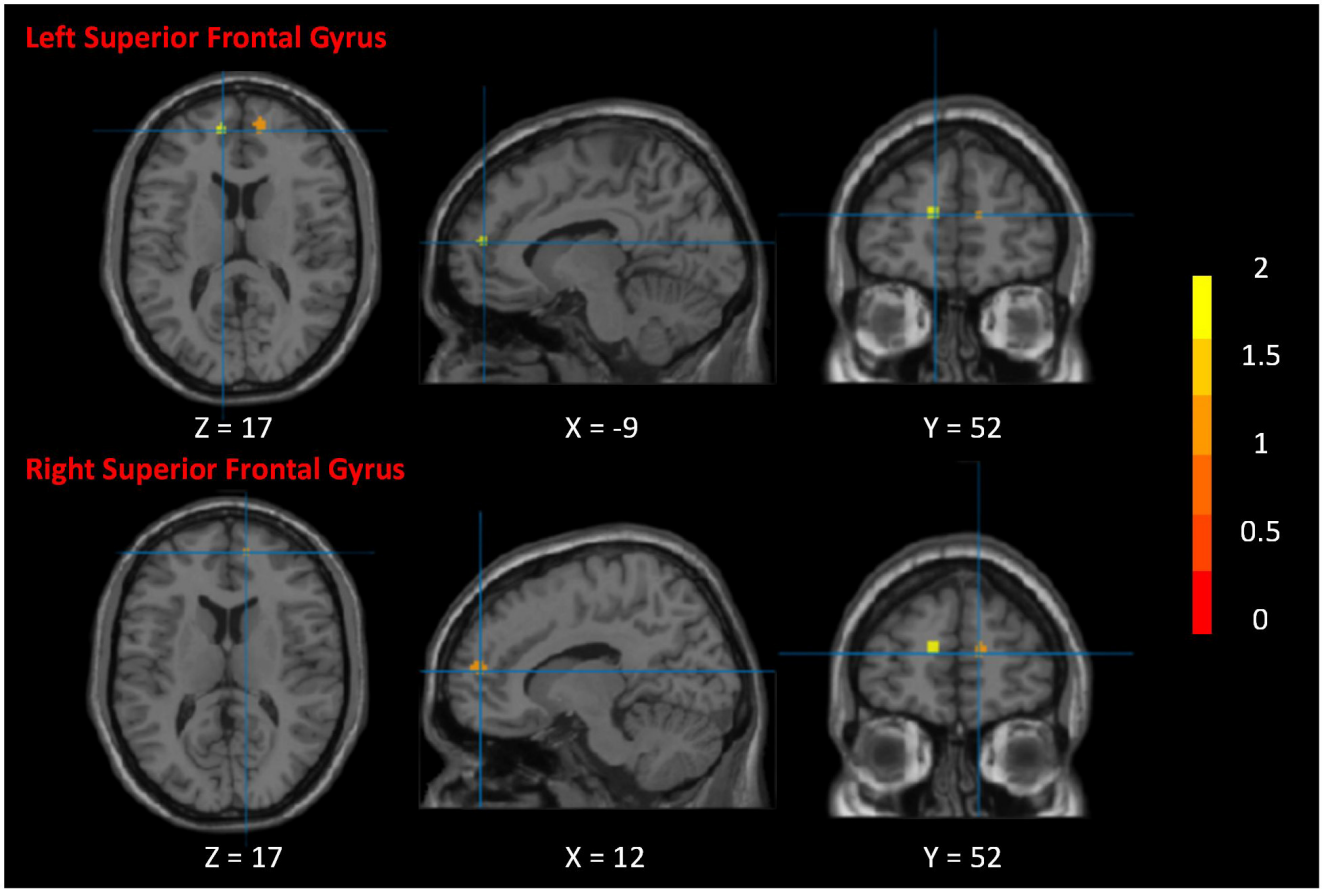
Neurological effects of Tai Chi Chuan. The figure shows the significant clusters of the activation likelihood estimated analysis. The color bar represents the *Z*-value of the clusters. The two clusters are the right superior frontal gyrus and left superior frontal gyrus.

After activation likelihood estimation, two clusters have undergone significant functional changes, including the right superior frontal gyrus (SFG.R) (centered at 12, 56, 15) and left superior frontal gyrus (SFG.L) (centered at –12, 54, 16). Cluster SFG.R has 296 *mm*^3^ volume, and cluster SFG.L has 192 *mm*^3^ volume (Table 2; Figure 4).

**Table 2:** Neurological effects of Tai Chi Chuan.

**Cluster**	***x***	***y***	***z***	**ALE-value**	***Z*-value**	**Volume**	**Atlas Label**
SFG.R	12	56	15	0.0217	5.29	296	Frontal Lobe: SFG, MFG
SFG.L	–12	54	16	0.0198	5.00	192	Frontal Lobe: SFG, MFG

ALE, activation likelihood estimated; SFG, superior frontal gyrus; MFG, medial frontal gyrus; L, left hemisphere; R, right hemisphere.

## Discussion

**Figure 5:**
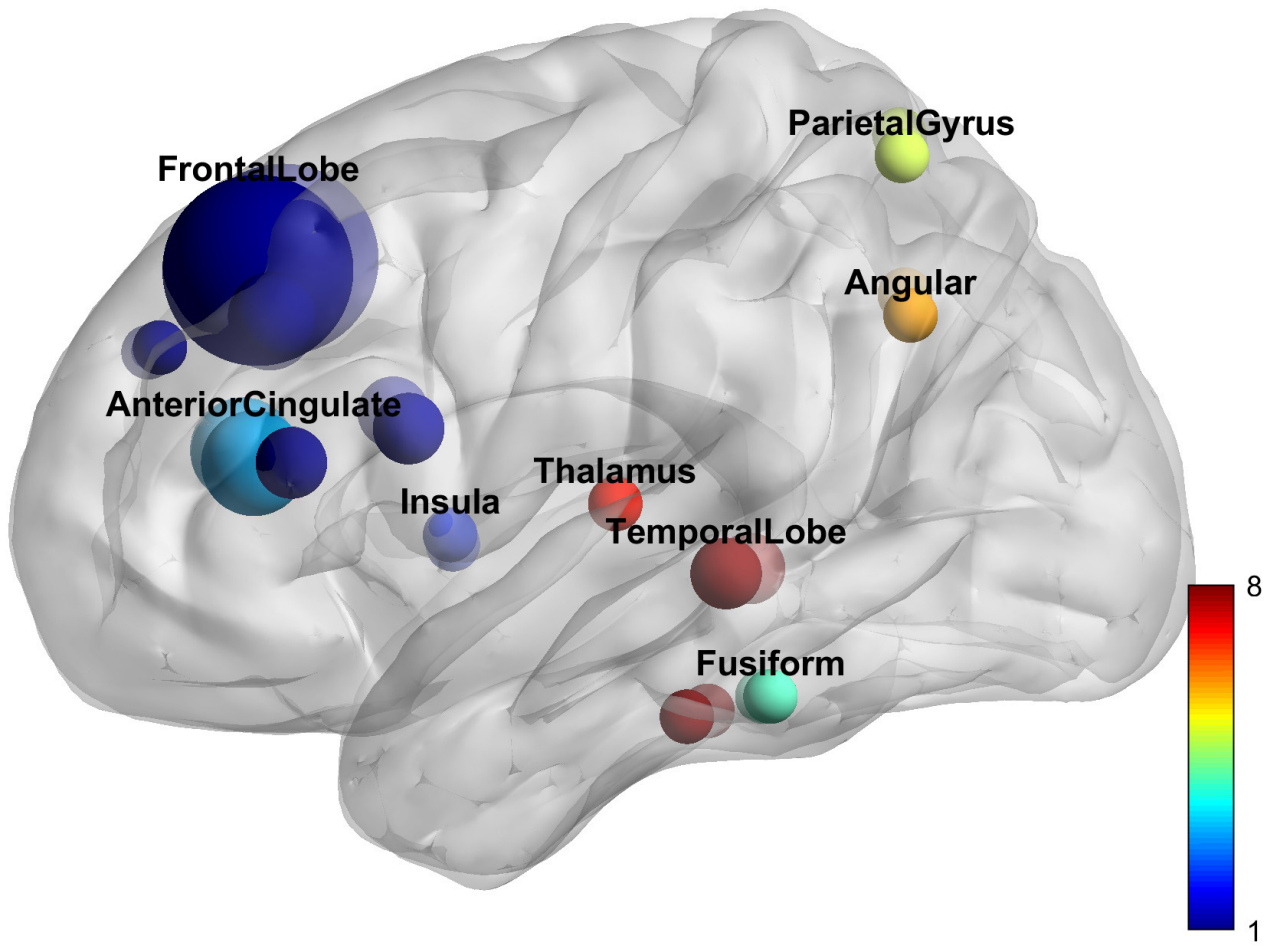
Reported brain regions.

We combed studies that used TCC as the primary intervention method with fMRI imaging. The search strategy resulted in 422 relevant articles, 18 of which were included in this meta-analysis. Six hundred seventy-seven subjects were enrolled in the current review. Among all study types, cross-sectional studies (8, 44%) and clinical control studies (7, 39%) were the most numerous. Among all subjects, healthy subjects were the majority (3, 61%). Disease-related studies included musculoskeletal diseases (4, 57%), anxiety and depression (2, 28%), and respiratory diseases (1, 15%). Yang-style 24-form TCC is the most common intervention; the average training course duration is 5 days/week for 12 weeks. Beck depression inventory score is the most common scale for evaluating cognitive function. Among all analysis methods, functional connectivity was the most common (10, 56%), followed by ALFF (3, 17%), and there were other analysis methods such as ReHo and VMHC. Regarding the reported brain regions, the most results came from the prefrontal lobe (12, 67%), followed by the cingulate cortex (4, 22%; Figure 5; Supplementary Table 2).

TCC research designs can generally be divided into two types. The first type investigates the neuromodulatory effects of TCC in resting state. These effects are typically categorized into two subtypes: (1) effects on healthy individuals and (2) effects on individuals with diseases. These studies focus on long-term effects and align with the traditional understanding of TCC, which suggests that its benefits require a sustained period to manifest. It is crucial for these studies to distinguish between the effects of TCC and conventional exercise. To achieve this, a control group should be included in the analysis, or the number of samples should be increased to ensure the collection of specific TCC effects. From a population perspective, TCC is generally more accepted among college students and the elderly. College students often engage in TCC to fulfill exercise requirements, while the elderly believe in its long-term health benefits. When selecting elderly participants, careful attention must be given to the collection of MRI data. Extremely old individuals or those with significant age disparities can lead to unstable MRI data. From an analytical standpoint, rs-fMRI analysis is the most common and accepted method. Another approach involves structural MRI analysis, which is often used in TCC research. Structural MRI analysis is based on the theory of neuroplasticity, and short-term exercise may not yield noticeable structural differences. Additionally, structural changes are highly sensitive to age variations; therefore, it is recommended to apply structural analysis mainly in healthy subjects.

Another approach to studying the neuromodulation effects of TCC is hypothesis-driven research. For example, it is hypothesized that TCC can regulate working memory. To test this hypothesis, an experimental paradigm related to working memory is required to detect changes following TCC practice. This type of research lacks a specific standard model and is susceptible to various factors, such as the mental state of the subjects. Therefore, it necessitates support from psychology-related backgrounds and the use of appropriate scales to assess mental states. Due to the limitations of MRI imaging equipment, analyzing neural activity during TCC is not feasible. Typically, this research employs functional near-infrared spectroscopy, which is less prone to interference and was not used in the present study. The methods for analyzing TCC's neuromodulatory effects based on task design are not standardized. For instance, block design can only analyze regional activation within a certain period, related to hemodynamic response functions. Event-related design provides valuable temporal information about neuronal activity but requires numerous experiments to achieve a sufficient signal-to-noise ratio and statistical power. Mixed designs, which combine elements of block and event-related designs, allow the study of both “maintained• and “transient• neural activities but present challenges in estimating the hemodynamic response function.

The findings indicate that cognitive control function constitutes a complex, high-order neural activity that involves the integration of multiple brain regions. This function is primarily responsible for the establishment of goals, the formulation of plans, the implementation of tasks, as well as the detection and maintenance of task-related activities (Botvinick and Braver, 2015). With advancing age, there is a tendency for a decline in executive control functions, including human working memory and attention. Additionally, there is a significant reduction in cognitive control functions and the functional connectivity between brain regions. These declines result in diminished consistency between goals and behavior and a reduction in executive control capabilities (Luna et al., 2010). Studies have shown that long-term regular TCC can effectively reshape the structure and functional activities of cognitive control brain areas in middle-aged and older adults and enhance the response to task stimuli and executive control abilities (Tao et al., 2017). They are reflected explicitly in positively improving the function of the prefrontal cortex and significantly reshaping the brain structure, especially the gray matter structure of multiple cognitive control brain areas such as the insula, hippocampus/parahippocampal gyrus, thalamus, anterior cingulate gyrus, and temporal lobe. Improve its functions, significantly improve response speed (Halassa and Kastner, 2017), reduce reaction time (Parent, 2016), and reduce the probability of errors during execution (Wu et al., 2019). Also, compared with healthy controls of the same age, the hippocampus and thalamus of the brains of long-term regular TCC exercisers, the volume of gray matter increases, and the thickness of the cortex of the anterior insula, precentral gyrus, middle frontal sulcus, superior temporal gyrus, medial occipitotemporal sulcus, and lingual sulcus increase (Wei et al., 2014).

According to our results, SFG is the most critical brain region in the neurological effects of TCC. The prefrontal cortex is the core brain area of the default mode network (Smallwood et al., 2021). It can widely receive and process information from other brain areas and promptly send regulatory instructions to different brain areas (Carlén, 2017). It has individual cognitive behavior (Hanganu-Opatz et al., 2023), decision-making, and memory (Euston et al., 2012). Complex cognitive functions such as encoding and retrieval and emotion perception play the role of the brain's center; the impact of many neurological diseases on the prefrontal cortex has also been confirmed (Xu et al., 2019). Research shows that TCC, as a complex exercise that integrates body movements, mind meditation (Deepeshwar et al., 2015), and breathing, has an upbeat positive regulatory effect (Moriarty et al., 2019) on the structure and function of the brain's prefrontal lobe. Long-term regular TCC can effectively improve the health of middle-aged and older adults (Silveira et al., 2019). It can improve cognitive ability, enhance memory and decision-making ability, reduce mood swings, effectively increase the volume of gray matter in the prefrontal lobe, and enhance the performance of spontaneous functional activities. This finding is particularly evident in patients with Alzheimer's disease and patients with mild cognitive impairment (Colangeli et al., 2016). Related studies have shown that these two groups' mental and memory abilities are associated with decreased prefrontal gray matter volume and decreased brain functional activity (Haeger et al., 2019). At the same time, the central nervous system regulation mechanism of TCC targets not only neurological diseases but also non-nervous system diseases, which can be improved in various ways through TCC. As a result, regular TCC effectively reshapes the structure and function of the prefrontal lobe, improves cognitive memory in middle-aged and older adults, and delays brain aging.

Despite the ALE results demonstrating significance in only two regions, the insights derived from other studies warrant consideration. Empirical research has demonstrated that regular engagement in Tai Chi Chuan (TCC) exercise substantially influences the brain functional activities of middle-aged and older adults. This includes enhancements in spontaneous functional brain activities and the synchrony of these activities (Wei et al., 2014). Furthermore, regular TCC exercise under task-specific conditions has been empirically validated to augment brain function in middle-aged and older adults when performing tasks (Wu et al., 2018). Specifically, following a 12-week regimen of traditional TCC, participants exhibited increased activation in the left superior frontal gyrus (SFG) and the right middle frontal gyrus during task execution. Correlation analyses revealed that alterations in functional activity signals within the left SFG and right middle frontal gyrus were negatively correlated with task-switching error rates. Additionally, another study indicated that the calcarine cortex, occipital cortex, and frontal pole exhibited significant activation in individuals with long-term regular TCC practice when engaged in attention tasks (Liu et al., 2018). These findings collectively suggest that regular TCC practice can effectively enhance spontaneous functional activities and the synchrony of brain functional activities and augment participants' cognitive memory, executive control, and emotional regulation functions.

Although the extensive body of fMRI literature, the studies analyzed in this investigation exhibited sample sizes considered minor by conventional standards and did not adhere to a rigorous sample size calculation formula (Szucs and Ioannidis, 2020). In the publication bias analysis, no correlation was observed between sample size and the number of reported foci, suggesting that the inflation of asserted foci may disproportionately impact smaller studies within the literature. TCC is an exercise modality widely practiced by individuals across diverse demographic groups. Regardless, the extant research does not focus on specific populations, necessitating reliance on studies encompassing healthy individuals and those with various pathologies. Subgroup analyses should have accounted for the heterogeneity in subject populations. Nevertheless, robust findings are precluded due to the limited number of studies concentrating on disease-specific populations (fewer than 10). This constraint may have influenced the outcomes of the ALE analysis, potentially leading to an overemphasis on the prefrontal cortex while overlooking regulatory effects originating from other brain regions. Furthermore, small-scale fMRI studies yielding inconclusive, invalid, or less promising results (e.g., few identified foci) may need to be published (Carp, 2012). From a study design perspective, researchers investigating TCC have often conceptualized it primarily as a medical intervention. However, compared to studies of pharmacological or other types of interventions, TCC training studies have reported limited items. Notably, aspects such as the evaluation of TCC training have yet to be consistently reported across studies. Another critical issue that requires addressing is the inconsistency in assessing subjects' mental states. Nearly half of the studies did not conduct evaluations of participants' mental conditions, with the Beck Depression Inventory score being the most frequently utilized assessment tool. When employing fMRI for research purposes, the influence of mental states on the results must be considered. Future studies should utilize a wide array of scales to evaluate distinct mental states comprehensively.

## Data Availability

The original contributions presented in the study are included in the article/Supplementary material, further inquiries can be directed to the corresponding authors.
