# Supplementary material for: Taichi on the brain: an activation likelihood estimated meta-analysis of functional neuroimaging data
Source: Front Hum Neurosci. 2025 Jan 22;18:1493677. doi: 10.3389/fnhum.2024.1493677 (PMC11794210; doi:10.3389/fnhum.2024.1493677)
Supplement: Supplementary file 1 [file Table_1.docx]

Supplementary Material

# Supplementary Table 1

| **Section and Topic** | **Item #** | **Checklist item** | **Location where item is reported** |
| --- | --- | --- | --- |
| **TITLE** | | |  |
| Title | 1 | Identify the report as a systematic review. | Title |
| **ABSTRACT** | | |  |
| Abstract | 2 | See the PRISMA 2020 for Abstracts checklist. | Abstract |
| **INTRODUCTION** | | |  |
| Rationale | 3 | Describe the rationale for the review in the context of existing knowledge. | The 3rd paragraph in introduction |
| Objectives | 4 | Provide an explicit statement of the objective(s) or question(s) the review addresses. | The 3rd paragraph in introduction |
| **METHODS** | | |  |
| Eligibility criteria | 5 | Specify the inclusion and exclusion criteria for the review and how studies were grouped for the syntheses. | Literature search and study selection section |
| Information sources | 6 | Specify all databases, registers, websites, organisations, reference lists and other sources searched or consulted to identify studies. Specify the date when each source was last searched or consulted. | Literature search and study selection section |
| Search strategy | 7 | Present the full search strategies for all databases, registers and websites, including any filters and limits used. | Literature search and study selection section |
| Selection process | 8 | Specify the methods used to decide whether a study met the inclusion criteria of the review, including how many reviewers screened each record and each report retrieved, whether they worked independently, and if applicable, details of automation tools used in the process. | Literature search and study selection section |
| Data collection process | 9 | Specify the methods used to collect data from reports, including how many reviewers collected data from each report, whether they worked independently, any processes for obtaining or confirming data from study investigators, and if applicable, details of automation tools used in the process. | Data extraction section |
| Data items | 10a | List and define all outcomes for which data were sought. Specify whether all results that were compatible with each outcome domain in each study were sought (e.g. for all measures, time points, analyses), and if not, the methods used to decide which results to collect. | Data extraction section |
|  | 10b | List and define all other variables for which data were sought (e.g. participant and intervention characteristics, funding sources). Describe any assumptions made about any missing or unclear information. | Data extraction section |
| Study risk of bias assessment | 11 | Specify the methods used to assess risk of bias in the included studies, including details of the tool(s) used, how many reviewers assessed each study and whether they worked independently, and if applicable, details of automation tools used in the process. | Literature Quality Evaluation section |
| Effect measures | 12 | Specify for each outcome the effect measure(s) (e.g. risk ratio, mean difference) used in the synthesis or presentation of results. | Activation likelihood estimation section |
| Synthesis methods | 13a | Describe the processes used to decide which studies were eligible for each synthesis (e.g. tabulating the study intervention characteristics and comparing against the planned groups for each synthesis (item #5)). | Activation likelihood estimation section |
|  | 13b | Describe any methods required to prepare the data for presentation or synthesis, such as handling of missing summary statistics, or data conversions. | Activation likelihood estimation section |
|  | 13c | Describe any methods used to tabulate or visually display results of individual studies and syntheses. | Activation likelihood estimation section |
|  | 13d | Describe any methods used to synthesize results and provide a rationale for the choice(s). If meta-analysis was performed, describe the model(s), method(s) to identify the presence and extent of statistical heterogeneity, and software package(s) used. | Activation likelihood estimation section |
|  | 13e | Describe any methods used to explore possible causes of heterogeneity among study results (e.g. subgroup analysis, meta-regression). | Activation likelihood estimation section |
|  | 13f | Describe any sensitivity analyses conducted to assess robustness of the synthesized results. | Activation likelihood estimation section |
| Reporting bias assessment | 14 | Describe any methods used to assess risk of bias due to missing results in a synthesis (arising from reporting biases). | Literature Quality Evaluation section |
| Certainty assessment | 15 | Describe any methods used to assess certainty (or confidence) in the body of evidence for an outcome. | Activation likelihood estimation section |
| **RESULTS** | | |  |
| Study selection | 16a | Describe the results of the search and selection process, from the number of records identified in the search to the number of studies included in the review, ideally using a flow diagram. | General Literature Information in Results |
|  | 16b | Cite studies that might appear to meet the inclusion criteria, but which were excluded, and explain why they were excluded. | General Literature Information in Results |
| Study characteristics | 17 | Cite each included study and present its characteristics. | General Literature Information in Results |
| Risk of bias in studies | 18 | Present assessments of risk of bias for each included study. | Literature Quality in Results |
| Results of individual studies | 19 | For all outcomes, present, for each study: (a) summary statistics for each group (where appropriate) and (b) an effect estimate and its precision (e.g. confidence/credible interval), ideally using structured tables or plots. | Table1 |
| Results of syntheses | 20a | For each synthesis, briefly summarise the characteristics and risk of bias among contributing studies. | Figure2 |
|  | 20b | Present results of all statistical syntheses conducted. If meta-analysis was done, present for each the summary estimate and its precision (e.g. confidence/credible interval) and measures of statistical heterogeneity. If comparing groups, describe the direction of the effect. | Figure3 |
|  | 20c | Present results of all investigations of possible causes of heterogeneity among study results. | Figure2 |
|  | 20d | Present results of all sensitivity analyses conducted to assess the robustness of the synthesized results. | N.A. |
| Reporting biases | 21 | Present assessments of risk of bias due to missing results (arising from reporting biases) for each synthesis assessed. | N.A. |
| Certainty of evidence | 22 | Present assessments of certainty (or confidence) in the body of evidence for each outcome assessed. | N.A. |
| **DISCUSSION** | | |  |
| Discussion | 23a | Provide a general interpretation of the results in the context of other evidence. | The 1st paragraph of Discussion |
|  | 23b | Discuss any limitations of the evidence included in the review. | The 5th paragraph of Discussion |
|  | 23c | Discuss any limitations of the review processes used. | The 5th paragraph of Discussion |
|  | 23d | Discuss implications of the results for practice, policy, and future research. | The 5th paragraph of Discussion |
| **OTHER INFORMATION** | | |  |
| Registration and protocol | 24a | Provide registration information for the review, including register name and registration number, or state that the review was not registered. | N.A. |
|  | 24b | Indicate where the review protocol can be accessed, or state that a protocol was not prepared. | N.A. |
|  | 24c | Describe and explain any amendments to information provided at registration or in the protocol. | N.A. |
| Support | 25 | Describe sources of financial or non-financial support for the review, and the role of the funders or sponsors in the review. | Additional information section |
| Competing interests | 26 | Declare any competing interests of review authors. | Additional information section |
| Availability of data, code and other materials | 27 | Report which of the following are publicly available and where they can be found: template data collection forms; data extracted from included studies; data used for all analyses; analytic code; any other materials used in the review. | Additional information section |

# Supplementary Table 2

| **Study ID** | **Comparison Type** | **Participation type** | **Sample Size** | **Gender (Male/Female)** | **Age (Years)** | **Education (Years)** | **Intervention type (Taichi style)** | **Intervention duration** | **Training experience of coaches (Years)** | **Mental status assessment** | **Analysis methods** | **Results (Brain Regions)** |
| --- | --- | --- | --- | --- | --- | --- | --- | --- | --- | --- | --- | --- |
| Tao, J. 2016 | clinical controlled trial | older adults | 21 | 8/13 | 62.38 ± 4.55 | 9.61 ± 3.02 | Yang-style 24-form TCC | 5 days/week for 12 weeks, for 60 min/session | 5 | MMSE, BDI | Seed-to-Voxel Analysis | mPFC |
| Tao, J. 2017a | clinical controlled trial | older adults | 21 | 8/13 | 62.38 ± 4.55 | 9.61 ± 3.02 | Yang-style 24-form TCC | 5 days/week for 12 weeks, for 60 min/session | 5 | MMSE, BDI | Seed-to-Voxel Analysis | SFG, dACC, rACC |
| Tao, J. 2017b | clinical controlled trial | older adults | 21 | 8/13 | 62.38 ± 4.55 | 9.61 ± 3.02 | Yang-style 24-form TCC | 5 days/week for 12 weeks, for 60 min/session | 5 | MMSE, BDI | amplitude of low frequency fluctuations Analysis | DLPFC |
| Liu, Z. 2018 | cross-sectional study | tai chi practitioners | 26 | 8/18 | 65.19 ± 2.30 | 10.46 ± 1.79 | N.A. | 66.76 ± 20.51 min/day | N.A. | BDI, NEO-FFI, FFMQ, MAAS | Seed-to-Voxel Analysis | Thalamus, Pallidum, MFG |
| Wu, M. 2018 | clinical controlled trial | older adults | 16 | 3/13 | 64.9 ± 2.8 | 13.8 ± 2.4 | Yang-style 24-form TCC | 3 days/week for 12 weeks, for 60 min/session | 10 | MoCA, CDR, GDS | BOLD response magnitude | SFG, MFG, IFG, IPG, AG |
| Cui, L. 2019 | cross-sectional study | college students | 12 | 2/10 | 21.83 ± 2.48 | 16.33 ± 2.23 | Bafa Wubu of Tai Chi | 3 days/week for 8 weeks, for 60 min/session | N.A. | - | Seed-to-Voxel Analysis | SPL |
| Kong, J. 2019 | cohort study | fibromyalgia | 21 | 1/20 | 53.10 ± 11.58 | N.A. | standardized Tai Chi protocol developed for patients with fibromyalgia | 2 days/week for 12 weeks, for 60 min/session | N.A. | BDI | Seed-to-Voxel Analysis | rACC, mPFC |
| Liu, J. 2019a | cross-sectional study | Knee osteoarthritis | 28 | 6/22 | 40-70 | N.A. | Yang-style 24-form TCC | 5 days/week for 12 weeks, for 60 min/session | 5 | BDI | Seed-to-Voxel Analysis | mPFC |
| Liu, J. 2019b | cross-sectional study | Knee osteoarthritis | 28 | 6/22 | 40-70 | N.A. | Yang-style 24-form TCC | 5 days/week for 12 weeks, for 60 min/session | 5 | BDI | Seed-to-Voxel Analysis | DLPFC |
| Liu, J. 2019c | clinical controlled trial | older adults | 21 | 8/13 | 62.38 ± 4.55 | 9.61 ± 3.02 | Yang-style 24-form TCC | 5 days/week for 12 weeks, for 60 min/session | 5 | BDI | Seed-to-Voxel Analysis | mPFC |
| Chen, L. 2020 | clinical controlled trial | older adults | 22 | 7/15 | 52.36 ± 6.88 | 12.18 ± 3.03 | Yang-style, Wu-style, Sun-style,and modified Chen-style TCC | N.A | N.A. | - | voxel-mirrored homotopic connectivity | PCG |
| Liu, Z. 2020 | clinical controlled trial | older adults | 31 | 10/21 | 64.93 ± 2.37 | 10.52 ± 1.91 | N.A. | 66.36 ± 21.96 min/day | N.A. | - | BOLD response magnitude Analysis | ACC, MTG |
| Xu, A. 2020 | cohort study | major depressive disorder | 16 | 6/10 | 46.5 ± 18.5 | N.A. | Yang-style 24-form TCC | 5 days/week for 10 weeks, for 40 min/session | 25 | HAMD | Seed-to-Voxel Analysis | INS |
| Yue, C. 2020a | cross-sectional study | elderly women | 20 | 0/20 | 62.90 ± 2.38 | 9.05 ± 1.80 | Yang-style 24-form TCC | 5 times/week, for 90 min/session | N.A. | MoCA | Regional Homogeneity Analysis | MTG, ITG |
| Shen, Q. 2021 | cross-sectional study | Young Adults | 12 | 2/10 | 21.83 ± 2.48 | 16.33 ± 2.23 | Bafa Wubu | 3 days/week for 8 weeks, for 60 min/session | N.A. | - | amplitude of low frequency fluctuations Analysis | SFG, fusiform gyrus |
| Shen, H. 2022 | cohort study | Chronic Obstructive Pulmonary Disease | 20 | 15/5 | 66.90 ± 7.17 | N.A. | Yang-style 24-form TCC | 3 days/week for 8 weeks, for 90 min/session | N.A. | - | Degree of Centrality | IFG |
| Zhang, J. 2023 | cross-sectional study | anxiety and depression | 9 | 2/7 | 24.20 ± 4.07 | N.A. | Bafa Wubu of Tai Chi | 5 days/week for 8 weeks, for 60 min/session | N.A. | SAS, SDS | amplitude of low frequency fluctuations Analysis | MFG, IOG, MTG |
| Liu, J. 2024 | cross-sectional study | Knee osteoarthritis | 28 | 6/22 | 40-70 | N.A. | Yang-style 24-form TCC | 5 days/week for 12 weeks, for 60 min/session | 5 | BDI | Seed-to-Voxel Analysis | mPFC |

Abbreviation, MMSE,Mini-Mental State Exam; BDI, Beck Depression Inventory; NEO-FFI, NEO Five-Factor Inventory; FFMQ, Five Facets Mindfulness Questionnaire; MAAS, Mindful Attention Awareness Scale; MoCA,Montreal Cognitive Assessment; CDR, Clinical Dementia Rating; GDS, Geriatric Depression Scale; HAMD, Hamilton Depression Rating Scale; SAS, Zung Self-Rating Anxiety Scale; SDS, Zung Self-Rating Depression Scale; mPFC, medial prefrontal cortex; dACC, dorsal anterior cingulate cortex; rACC, rostral anterior cingulate cortex; DLPFC, dorsolateral prefrontal cortex; MFG, middle frontal gyrus; SFG, superior frontal gyrus; IFG, inferior frontal gyrus; IPG, inferior parietal gyrus; AG, angular gyrus; PCG, precentral gyrus; ACC, anterior cingulate cortex; MTG, middle temporal gyrus; INS, insula cortex; ITG, inferior temporal gyrus; IOG, inferior occipital gyrus.
